# TMT-based quantitative proteomics reveals the targets of andrographolide on LPS-induced liver injury

**DOI:** 10.1186/s12917-023-03758-2

**Published:** 2023-10-10

**Authors:** Shihao Ge, Wenqi Lian, Yongjiang Bai, Linzheng Wang, Fuwei Zhao, Houmei Li, Dongliang Wang, Quanhai Pang

**Affiliations:** 1 College of Veterinary Medicine, Shanxi Agricultural University, Taigu, 030801 Shanxi China; 2 College of Pharmacy, Heze University, Heze, 274000 Shangdong China; 3 College of Traditional Chinese Medicine, Shandong University of Traditional Chinese Medicine, Jinan, 250035 Shangdong China; 4 Shuozhou grass and animal husbandry development center, ShuoZhou, 036000 Shanxi China; 5 ShuoZhou Vocational Technology College, ShuoZhou, 036000 Shanxi China

**Keywords:** Proteomics, Andrographolide, LPS, Liver injury, Targets

## Abstract

**Background:**

Andrographolide (Andro) is a diterpenoid derived from Andrographis paniculate, which has anti-inflammatory, antibacterial, antiviral and hepatoprotective activities. Gram-negative bacterial infections can cause varying degrees of liver injury in chickens, although Andro has been shown to have a protective effect on the liver, its underlying mechanism of action and effects on liver proteins are not known.

**Methods:**

The toxicity of Andro on the viability of leghorn male hepatoma (LMH) cells at different concentrations and times was analyzed by CCK-8 assays. Alanine aminotransferase (ALT) and aspartate aminotransferase (AST) activities in the culture supernatants were measured using an automatic biochemical analyzer to evaluate the protective effect of androscopolide on LPS-induced injury of LMH cells. Subsequently, TMT proteomics analysis were performed on the negative control group (NC group), LPS, and LPS-Andro groups, and bioinformatics analysis was performed on the differentially expressed proteins (DEPs).

**Results:**

It was found that Andro reduced ALT and AST levels in the cell supernatant and alleviated LPS-induced injury in LMH cells. Proteomic analysis identified 50 and 166 differentially expressed proteins in the LPS vs. NC group and LPS-Andro vs. LPS group, respectively. Andro may be involved in steroid metabolic processes, negative regulation of MAPK cascade, oxidative stress, and other processes to protect against LPS-induced liver injury.

**Conclusions:**

Andro protects against LPS-induced liver injury, HMGCS1, HMGCR, FDPS, PBK, CAV1, PRDX1, PRDX4, and PRDX6, which were identified by differential proteomics, may be the targets of Andro. Our study may provide new theoretical support for Andro protection against liver injury.

**Supplementary Information:**

The online version contains supplementary material available at 10.1186/s12917-023-03758-2.

## Background

The liver is essential for biotransformation and toxin removal [1]. Gram-negative bacteria are common pathogens that infect chickens, leading to decreased productivity and even death of the chickens, with severe losses to the chicken industry. Studies have found that many Gram-negative bacteria can cause varying degrees of liver injury in chickens [2–4]. Lipopolysaccharide (LPS) forms part of the cell walls of Gram-negative bacteria, where it interacts with the host during the infection process [5]. Pathogen pattern recognition receptors (PRRs) on the host cell surfaces recognize LPS, triggering an innate immune response by activating signaling pathways and stimulating pro-inflammatory cytokine production to defend against the bacterial invasion [6]. Toll-like receptors (TLRs) are a type of PRR and TLR4 is especially involved in the response to LPS. TLR4 recognizes LPS to activate mitogen-activated protein kinase (MAPK), leading to an up-regulation of inflammatory mediators, including tumor necrosis factor-α (TNF-α), and potentially causing inflammatory liver injury [7]. Furthermore, LPS can stimulate the production of reactive oxygen species (ROS) which, if not removed by the antioxidant defense system can lead to oxidative stress [8]. Studies have found that inflammation is usually accompanied by the formation of ROS and oxidative stress [9], and both are closely involved in inflammatory liver injury. Therefore, oxidative stress is considered to mediate the development of liver injury [10].

Traditional Chinese medicine (TCM) targets multiple pathways and proteins in the treatment of disease, using complex and diverse mechanisms of action. Proteomics provides a powerful means of studying the possible mechanisms of TCM, and many researchers have studied the potential targets of TCM in different diseases using proteomics technology [11–13]. Thus, proteomics is helpful to identify the potential target proteins or biomarkers associated with TCM treatment. Andrographolide (Andro) is a diterpenoid derived from *Andrographis paniculate* and is documented to have a variety of physiological effects, including anti-inflammatory, antiviral, antitumor, and immune regulatory properties [14]. Andro is also effective for hepatoprotection [15] and reduces the levels of alanine aminotransferase (ALT), aspartate aminotransferase (AST), interleukin (IL)-1β, and TNF-α in liver tissue by inhibiting nuclear factor-Kappa B (NF-κB) and activating the nuclear factor erythroid 2-related factor 2 (Nrf2) signaling pathway, thus protecting against liver damage resulting from by D-GalN/LPS treatment in mice [16]. Andro can also protect against cholestatic liver damage by reducing oxidative stress and inflammation [17]. However, Andro-mediated protection against LPS-induced liver damage has not been investigated using proteomics.

In this study, based on tandem mass tag (TMT) proteomics, we investigated the effects of Andro on LPS-induced liver damage in immortalized chicken leghorn male hepatoma (LMH) cells. Differentially expressed proteins (DEPs) were identified and further analyzed by Gene Ontology (GO) and Kyoto Encyclopedia of Genes and Genomes (KEGG) to gain insight into potential targets of Andro for protection against LPS-induced liver damage.

## Results

### Toxicity of Andro on the viability of LMH cells

**Fig. 1:**
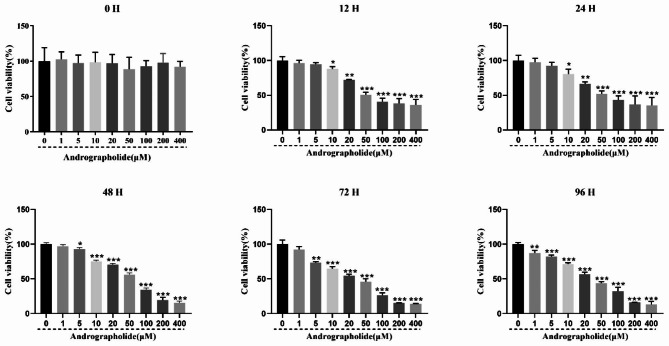
The cell viability of Andro on LMH cells. LMH cells were treated with different concentrations of Andro (0, 1, 5, 10, 20, 50, 100, 200 and 400 µM) for different time points (0, 12, 24, 48, 72 and 96 h), cell viability was measured using the CCK8 assay. (* *P* < 0.05, ** *P* < 0.01, *** *P* < 0.001 vs. 0 µM)

CCK-8 assays confirmed that the concentrations of Andro used for experiments did not reduce the viability of the cells. As shown in Fig. 1, the Andro concentrations used were 0, 1, 5, 10, 20, 50, 100, 200, and 400 µM, with times of 0, 12, 24, 48, 72, and 96 h. Andro was found to lower the viability of LMH cells in a time- and dose-dependent manner, with no significant differences seen between the different Andro concentrations at 0 h (*P* > 0.05). 12, or 24 h, Andro was found to be cytotoxic at concentrations greater than 10 µM. Therefore, concentrations of 1–5 µM which showed less cytotoxicity were selected for the following experiments.

### Influence of Andro on ALT and AST in LMH culture supernatants after LPS challenge

**Fig. 2:**
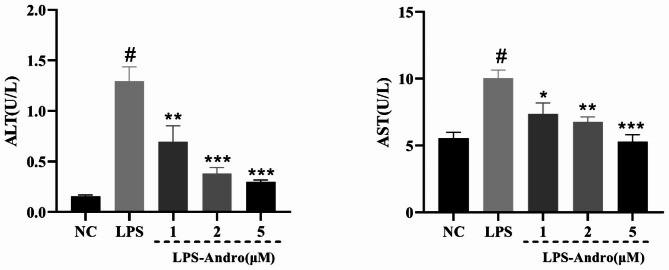
Effects of Andro on LPS-induced LMH cells supernatant AST (**A**) and ALT (**B**) levels. # *P* < 0.001 vs. NC, * *P* < 0.05, ** *P* < 0.01, *** *P* < 0.001 vs. LPS

ALT and AST activities are frequently used to indicate liver injury in clinical practice. Significant increases in both ALT and AST were observed after 24-h LPS challenge in LMH cells (*P* < 0.001), with different concentrations of Andro (1, 2, and 5 µM) leading to gradual reductions in ALT and AST activities and reaching significance (*P* < 0.001) at 5 µM Andro (Fig. 2). Therefore, 24 h, 5 µM Andro was used for proteomic assays.

### Differences in protein expression induced by LPS and andro

**Fig. 3:**
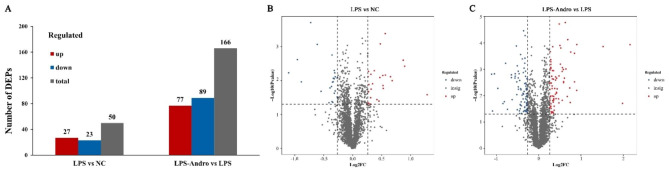
The number of significant differentially expressed proteins in 3 group. (**A**) The number of significant differentially expressed proteins between in LPS vs. NC group and LPS-Andro vs. LPS group (Blue represents down-regulated proteins, red represents up-regulated proteins, and gray represents total proteins); (**B**) Volcano plot of proteins identified from LPS and NC group; (**C**) Volcano plot of proteins identified from LPS-Andro and LPS group. (The abscissa of the volcano plot represents log2 of the Fold Change, the ordinate represents -log10 *P* value, red and blue scatter points represent up-regulated and down-regulated proteins, respectively, and gray represents non-significantly expressed proteins)

DEPs were screened between the LPS vs. NC groups and the LPS-Andro vs. LPS groups, using the thresholds of *P* < 0.05 and Fold Change (FC) > 1.2 or < 1/1.2. As shown in Fig. 3A-C, LPS significantly altered the protein levels in the LMH cells, compared with the NC group. Altogether, 50 DEPs were identified in the LPS group, of which 27 were up-regulated and 23 were down-regulated. Andro treatment significantly changed the numbers of DEPs, with 166 DEPs identified in comparison with the LPS group, of which 77 were up-regulated and 89 were down-regulated. The detailed information of DEPs is listed in Supplementary Table 1.

### Subcellular localization of the DEPs

**Fig. 4:**
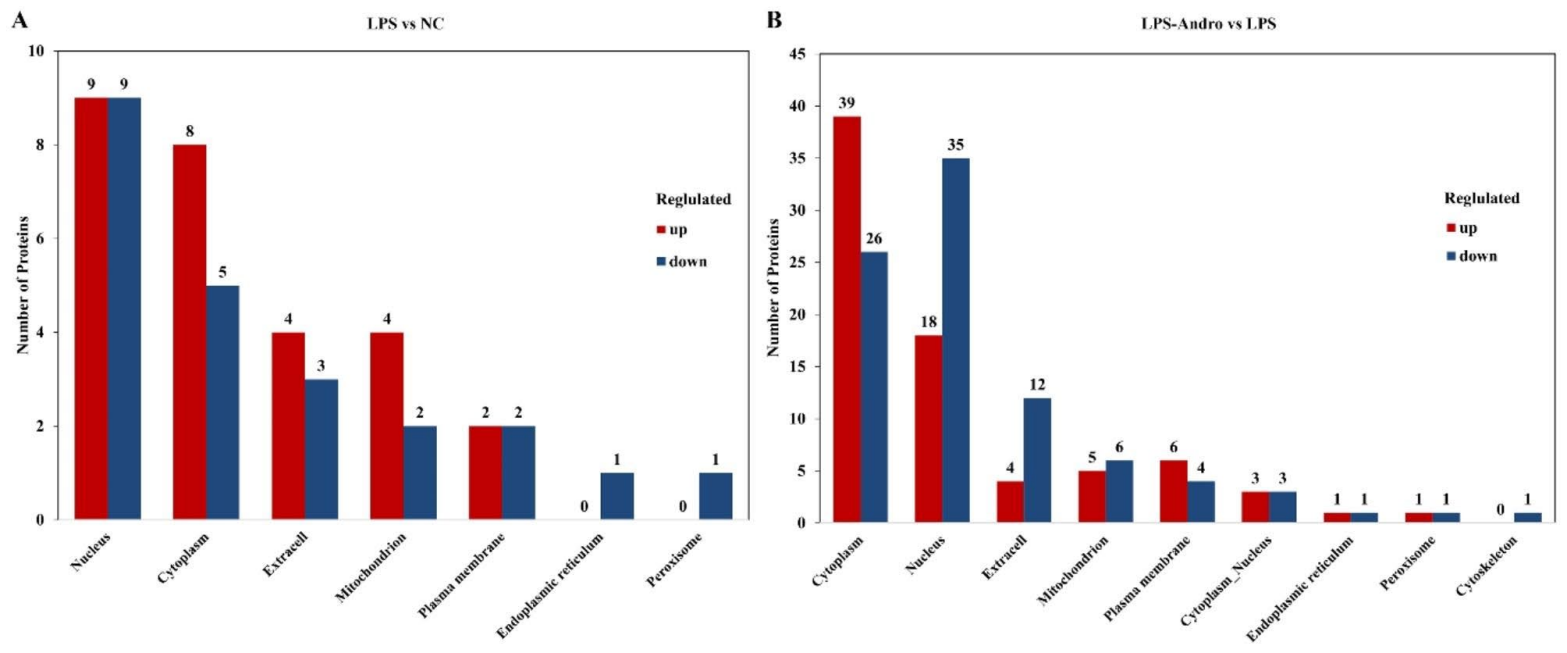
Subcellular localization of differential proteins, the abscissa represents the subcellular fraction, the ordinate represents the number of differential proteins, and the red and blue represent up-regulated and down-regulated differential proteins, respectively. (**A**) LPS vs. NC; (**B**) LPS-Andro vs. LPS

The subcellular localizations, as well as the numbers of up-and down-regulated proteins, were analyzed using PSORTb and WoLF PSORT soft, respectively. Figure 4 A showed the subcellular localizations of 27 proteins that were up-regulated and 23 that were down-regulated after LPS treatment. Of the 27 up-regulated proteins, nine were nuclear, eight were cytoplasmic, four were extracellular, and four were mitochondrial. Of the 23 down-regulated proteins, nine were nuclear, five were cytoplasmic, three were extracellular, and two were mitochondrial. Of the proteins expressed differentially between the LPS-Andro and LPS groups, as shown in Fig. 4B, the 77 up-regulated proteins were expressed predominantly in the cytoplasm, with 39 proteins, followed by the nucleus with 18, the mitochondria with five, and the extracellular environment with four proteins. Of the 89 down-regulated proteins, 35 were nuclear, 26 were cytoplasmic, 12 were extracellular, and six were mitochondrial.

### GO analysis of DEPs

**Fig. 5:**
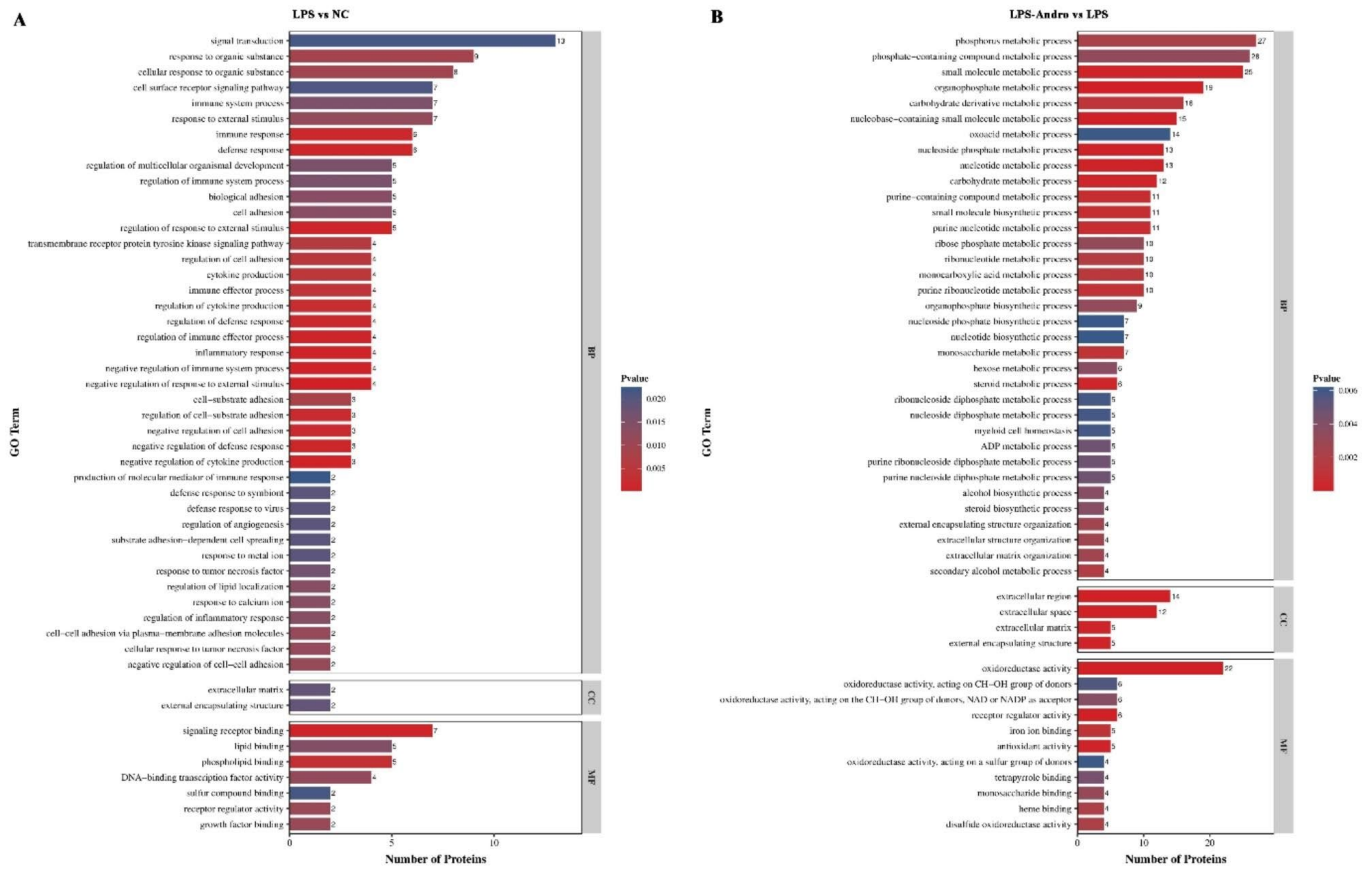
GO enrichment results of the DEPs, the abscissa represents the number of differentially annotated proteins, the ordinate represents the enriched GO entries, and the color change from blue to red represents the change of P-value from large to small. (BP: biological process; CC: cell component; MF: molecular function). (**A**) LPS vs. NC; (**B**) LPS-Andro vs. LPS

The DEPs were classified into the biological process (BP), cellular component (CC), and molecular function (MF) categories according to their GO annotations. As illustrated in Fig. 5A, LPS-treated cells, compared with the NC group, showed enrichment in the BP category, including processes such as signal transduction, immune response, responses to external stimuli, and inflammatory response. In the MF category, the DEPs between these two groups were mostly involved in signal receptor binding, lipid binding, and phospholipid binding. Figure 5B shows the comparison between the LPS and LPS-Andro groups; it can be seen that Andro induced the expression of proteins in the BP category related to phosphorus, carbohydrate, and steroid metabolism, as well as negative regulation of MAPK cascade, amongst others. In the MF category, the DEPs were mostly connected to oxidoreductase activity, receptor regulation, and antioxidant activity.

### KEGG pathway analysis of the DEPs

**Fig. 6:**
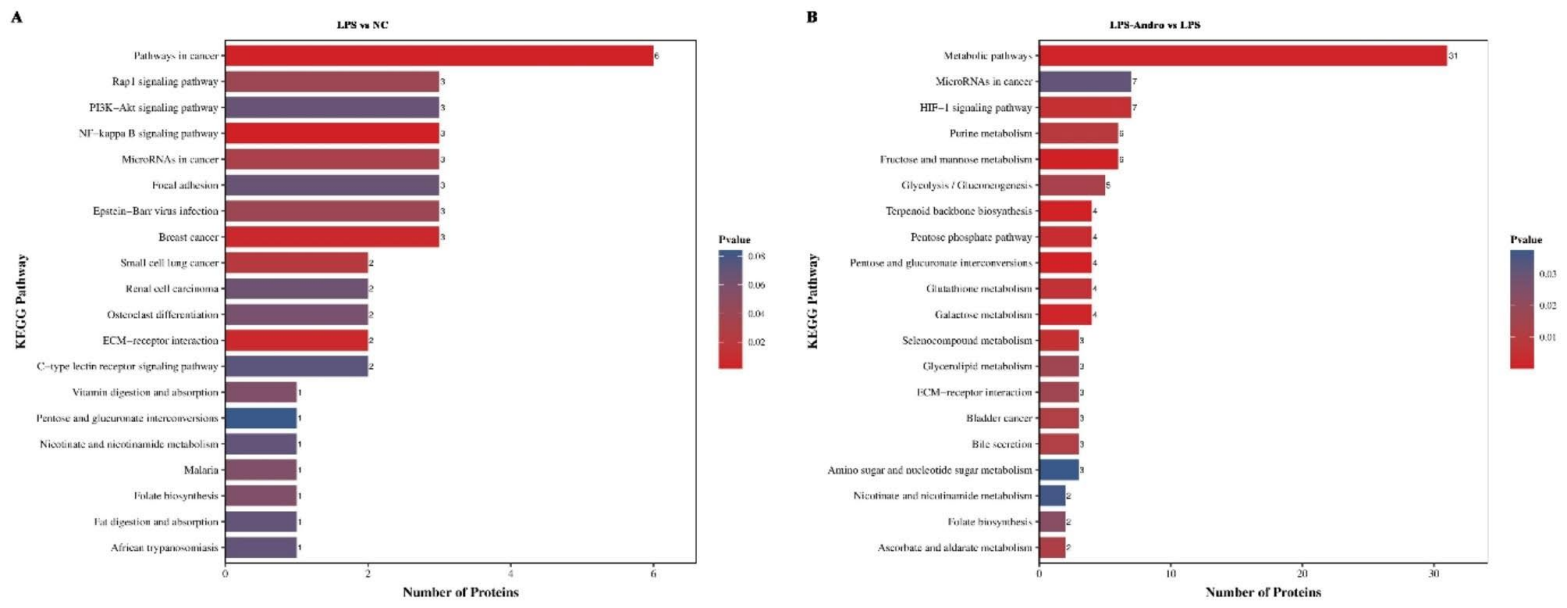
KEGG enrichment results of the DEPs, the abscissa represents the number of differentially annotated proteins, the ordinate represents the enriched KEGG pathway, and the color change from blue to red represents the change of P-value from large to small. (**A**) LPS vs. NC; (**B**) LPS-Andro vs. LPS

Further exploration of the signaling pathways underlying the effects of LPS and Andro was conducted by KEGG analysis of enriched pathways. The 50 DEPs identified after LPS treatment, compared with NC cells, were associated with 92 KEGG pathways, including the Rap1, phosphatidylinositol 3-kinase (PI3K)-protein kinase B (AKT), NF-κB, and MAPK signaling pathways (Fig. 6A). Compared with the LPS group, the 166 DEPs identified in the LPS-Andro group were involved in 223 KEGG pathways, with those of interest including pathways associated with metabolism and hypoxia-inducible factor-1 (HIF-1) signaling (Fig. 6B).

### PPI analysis of DEPs

**Fig. 7:**
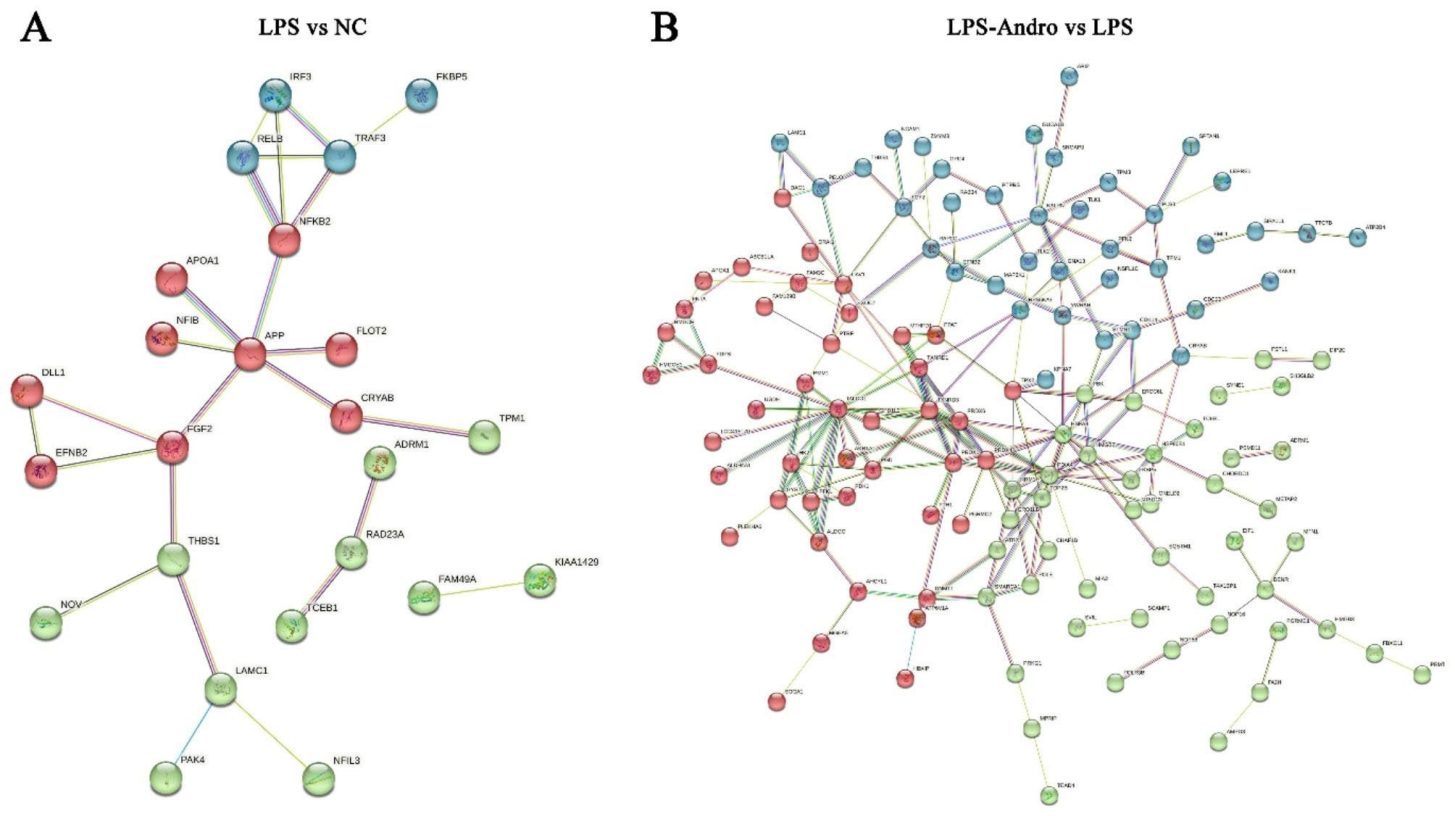
Protein-protein interaction networks built on STRING for the identified proteins. (**A**) LPS vs. NC; (**B**) LPS-Andro vs. LPS, different colors represent the three clusters distinguished by the K-means method

Using the StringDB database, we constructed a PPI network for the identified DEPs. Our results showed that App, NFκB2, RELB, TRAF3, and FGF2 played vital roles in the network after LPS treatment, as shown in Fig. 7A. After Andro treatment, the major proteins were PRDX1, PRDX4, PRDX6, PBK, and CAV1 (see Fig. 7B), which may be associated with oxidative stress and MAPK signaling.

## Discussion

The innate immune system is the main defense against microorganisms, and LPS can act as an inducer of innate immunity to activate the adaptive immune system [18]. LPS binds to the PRR TLR4 to activate MAPK and NF-κB signaling [19, 20], as well as the PI3K-AKT pathway [21], generating an inflammatory response. Consistent with this, the KEGG analysis indicated that LPS activated Rap1, PI3K-Akt, NF-κB, and MAPK signaling in LMH cells. GO analysis of the 50 DEPs between the LPS and NC groups indicated that they tended to be involved in biological processes such as the immune and inflammatory responses and responses to external stimuli such as TRAF3 and RELB. Proteins belonging to the tumor necrosis factor receptor (TNF-R)-associated factor (TRAF) family function in a variety of signal transduction pathways leading to the expression of a variety of immune receptors, including innate and adaptive and cytokine receptors [22]. TRAF3 signaling stimulates the NF-κB and MAPK pathways, both of which regulate inflammation and the inflammatory response [23, 24]. RELB, a member of the NF-κB transcription factor family, is significantly up-regulated during inflammation, which induces the formation of the RELB/p50/IκBα complex [25]. It was found that LPS caused upregulation of TRAF3 in human bronchial epithelium (16HBE) [26] and also increased RELB expression in RAW 264.7 cells [27]; our results are in agreement with these findings.

The 166 DEPs identified after Andro treatment, compared with cells treated only with LPS, were found to be involved in 223 KEGG pathways, with significant enrichment seen in pathways associated with metabolism and HIF-1 signaling. GO analysis indicated DEP involvement in biological processes such as steroid and carbohydrate metabolism and the negative regulation of MAPK cascades. HIF is a heterodimeric complex composed of α and β subunits, with the α subunit having two forms, HIF-1α and HIF-2α. HIF-1α is a key regulator of both cell metabolism and inflammation [28, 29], and LPS can activate the expression of HIF-1α [30]. Studies have shown that there are close relationships between HIF-1α, inflammation, and cholesterol metabolism [31–34]. Cholesterol accumulation in macrophages can augment the inflammatory response [32], promoting HIF-1 activation, and may mediate liver injury [33]. Some TCM can inhibit inflammation by interfering with HIF-1α; for example, curcumin can reduce inflammation and apoptosis by inhibiting HIF-1α expression and the total cholesterol content in macrophages [35]. Our results showed that Andro down-regulated the expression of HMGCS1, HMGCR, and FDPS involved in steroid metabolism. The mevalonate pathway is involved in lipid metabolism and is a key regulatory step in the de novo synthesis of cholesterol, with 3-hydroxy-3-methylglutaryl coenzyme A synthase 1(HMGCS1) and 3-hydroxy-3-methylglutaryl-coenzyme A reductase (HMGCR) playing key roles. HMGCS1 is a cytoplasmic enzyme upstream of HMGCR in the mevalonate pathway and can condense acetyl-CoA and acetoacetyl-CoA into HMG-CoA [36], a key enzyme in the biosynthesis of liver cholesterol. HMGCR is a rate-limiting enzyme in cholesterol synthesis, and increased HMGCR activity increases cholesterol synthesis in the liver. It has been found that inhibitors of HMGCS1 and HMGCR reduce liver cholesterol synthesis, thereby protecting against liver injury [37–39]. HMGCR appears to have a functional relationship with HIF-1α, as altering the expression of HIF-1α influences HMGCR expression in zebrafish [34], and HIF-1α can mediate the transcription of insulin-inducible gene 2 (Insig-2) leading to HMGCR degradation in the liver [40]. Another key enzyme in the mevalonate pathway, farnesyl diphosphate synthase (FDPS), is involved in the conversion of acetyl-CoA to cholesterol [41]; FDPS inhibitors, such as the bisphosphonate alendronate, reduce cholesterol synthesis and are used in the treatment of hypercholesterolemia [42, 43]. Polygala tenuifolia (PTE) down-regulates genes involved in lipid and cholesterol biosynthesis, including FDPS, to reduce lipid accumulation in an obese mouse model [44]. Our experimental results showed that Andro may inhibit the synthesis of cholesterol by down-regulating the expression of HMGCS1, HMGCR, and FDPS, thus inhibiting the inflammatory reaction and having a protective effect on liver injury. HMGCS1, HMGCR, and FDPS may thus be drug targets for the Andro inhibition of liver injury.

The MAPKs are a group of related serine-threonine protein kinases, and the MAPK cascade plays a key role in a wide variety of cellular activities, including proliferation, differentiation, apoptosis, oxidative stress, and inflammatory responses [45, 46]. Numerous studies have demonstrated a link between MAPK signaling and inflammation in many diseases [47], and the activation of the ERK1/2, JNK, and p38 pathways can increase the levels of pro-inflammatory factors, such as TNF-α, IL-1β, IL-6, and IL-8 [48, 49]. MAPK signaling is activated in LPS-induced liver injury [50–52]; thus, MAPK inhibition is closely involved in preventing and treating liver injury. While research has shown that Andro can inhibit inflammation through the MAPK pathway, most studies have focused on the roles of JNK, ERK1/2, and p38 in classic MAPK signaling [53, 54]. Here, the proteomic results indicated that Andro negatively regulates the MAPK cascade and down-regulates the expression of PBK and CAV1. PDZ-binding kinase (PBK, also known as TOPK) is a serine-threonine kinase belonging to the MAPK-kinase (MAPKK) family, that promotes phosphorylation of p38 MAPK [55], leading to the identification of p38 MAPK as a specific substrate of PBK [56] and suggesting the cell-type-dependent involvement of PBK in ERK/MAPK, p38 MAPK, and JNK signaling [57]. PBK can regulate cell survival, proliferation, growth, apoptosis, and inflammation [58, 59]. It has been observed that LPS increases PBK levels in leukemia cells while up-regulating the expression of inducible nitric oxide synthase (iNOS), suggesting that PBK may be involved in the inflammatory response or in inflammation-related diseases [60]. Paeonol-based derivatives can inhibit skin inflammation by inhibiting PBK-p38/JNK signaling pathway and down-regulating nitric oxide content in LPS-induced RAW264.7 cells [61]. Caveolin 1 (Cav1) is a structural protein of cell membranes and plays a role in regulating cholesterol distribution, inflammatory signal transduction, and other biological processes [62, 63]. Cav1 is involved in hepatocellular carcinoma and hepatocellular differentiation through its activation of MAPK signaling [64, 65], and inhibition of Cav1 is associated with the anti-inflammatory effects of some drugs [66–68]. Our experimental results showed that Andro may negatively regulate the MAPK cascade by down-regulating the expression of PBK and CAV1, thereby inhibiting MAPK signaling. These findings provide a new idea for Andro reduces inflammation through the MAPK pathway.

There is normally a balance between the production of ROS and reactive nitrogen species (RNS) and oxidant scavenging in the body, and low ROS/RNS levels are essential for cell signaling and the maintenance of cell homeostasis [69]. Oxidative stress results from an altered balance between ROS and/or RNS production and the antioxidant defense capabilities [70]. The production of ROS is an important factor in liver injury [71, 72], and ROS can affect cellular structures such as hepatocyte proteins, lipids, and DNA, leading to liver injury [73]. LPS-induced liver injury is often accompanied by oxidative stress [52, 71], and inhibition of oxidative stress and inflammation can alleviate LPS-induced liver injury [74]. Antioxidants are, therefore, considered to be promising treatments for liver injury [75]. The peroxiredoxins (PRDXs) are a ubiquitous family of antioxidant enzymes, with six different PRDXs (PRDX1-PRDX6) currently known [76]. PRDXs can catalyze the breakdown of H_2_O_2_ and alkyl peroxides to eliminate intracellularly generated ROS, thereby protecting cells against oxidative stress [77]. Andro is a natural antioxidant that scavenges ROS, inhibits free radical-producing enzymes, and protects the mitochondria, thereby treating diseases caused by oxidative stress [14]. We found that Andro up-regulated PRDX1, PRDX4, and PRDX6 and that they formed major components in the PPI networks. It has been found that PRDX1 reduces ROS levels and inhibits apoptosis induced by NF-κB or MAPK signaling [78, 79], and PRDX1 can interact with pro-Caspase-1 (CASP1) to block the assembly of the NLRP3 (NOD-, LRR-, and pyrin-domain containing protein 3) inflammasome complex, thus acting as a negative regulator of NLRP3 inflammasome activation [80]. PRDX4 is a secreted enzyme responsible for ROS scavenging both intracellularly and extracellularly and has a protective effect in cholestatic liver injury [81]. PRDX6 is a widely expressed antioxidant non-selenium glutathione peroxidase implicated in a variety of cellular activities [82]. PRDX6 protects against neuronal death caused by oxidative stress [83], and can reduce LPS-induced renal ROS concentrations and inactivate the p38 MAPK and JNK pathways, thereby attenuating LPS-induced acute kidney injury [84]. Therefore, Andro may inhibit oxidative stress by up-regulating the expression of PRDX1, PRDX4, and PRDX6, protecting against LPS-induced liver damage.

## Conclusion

Andro protects against LPS-induced liver injury. Proteomic analysis showed that this effect was mediated by the inhibition of steroid metabolism, negative regulation of MAPK signaling, and reducing oxidative stress. HMGCS1, HMGCR, FDPS, PBK, CAV1, PRDX1, PRDX4, and PRDX6 may be the targets of Andro.

### Methods

#### Drugs and reagents

Andro (98% purity) was obtained from Xi’an XiaoCao Plant Technology Co., Ltd. (Xi’an, China), LMH cells were obtained from the ATCC (No. CRL-2117), while LPS (Escherichia coli, serotype 055: B5) and Dimethyl Sulphoxide (DMSO) were from Sigma (St. Louis, MO, USA), Cell Counting Kit-8 (CCK-8) was from Dojindo Laboratories (Kamimashiki-gun, Kumamoto, Japan), and RPMI-1640 and fetal bovine serum (FBS) were from Gibco (Waltham, MA, USA).

### Cell culture and cell proliferation-toxicity assays

LMH cells were cultured in RPMI-1640 with 10% FBS and 1% PS (penicillin-streptomycin) at 37 ℃, 5% CO_2,_ and saturated humidity. The cells were harvested for subsequent experiments when they had reached a density of at least 1 × 10^4^/cm^2^.

The cytotoxicity of Andro to LMH cells was quantified with CCK-8 assays. LMH cells (1 × 10^5^/mL) were inoculated into 96-well plates (NEST, Hong Kong, China) and grown for 24 h at 37 ℃. Next, fresh RPMI-1640 medium containing different concentrations of Andro (0, 1, 5, 10, 20, 50, 100, 200, 400 µM) and 0.1% DMSO was added to each well, with three replicates used for each concentration. After incubation for 0, 12, 24, 48, 72, and 96 h, 10 µl CCK-8 solution was added to each well and allowed to incubate for 2 h at 37 ℃, after which the optical densities (OD values) at 450 nm were read in a microplate reader (Varioskan LUX, Thermo, USA).

### Effects of Andro on ALT and AST in LMH cells

NC group was treated with 0.1% DMSO and the LPS group was treated with 0.1% DMSO and 10 µg/ml LPS for 24 h [85]. Cells in the LPS-Andro group were pretreated with 1, 2, and 5 µM Andro and 0.1% DMSO for 1 h, followed by treatment with 10 µg/ml LPS for 24 h, with three replicates in each group. Lastly, ALT and AST activities in the culture supernatants were measured using an automatic biochemical analyzer (Chemray 240, Shenzhen Rayto Life and Analytical Sciences Co., Ltd. China).

### Sample preparation

The proteomic analysis included three groups, namely, the NC, LPS, and LPS-Andro groups. Cells were lysed with 8 M Urea/100 mM Tris-Cl and ultrasonication. Dithiothreitol (DTT, 10 mM) was added and incubated at 37 ℃ for 1 h, followed by alkylation with 40 mM iodoacetamide (30 min, room temperature in the dark). Protein concentrations were determined using the Bradford method. The urea solution was reduced to below 2 M by dilution with 100 mM Tris-HCl, and the proteins were digested at a 1:50 trypsin:protein (w/w) ratio overnight at 37 °C. Digestion was halted with TFA and the samples were centrifuged (12,000×g, 15 min). The supernatants were retained and desalinated using SEP-PAK C18, after which the material was drained and stored at -20 °C.

Isobaric TMT was used for DEP identification. TMT labeling was conducted in accordance with the provided directions. Specifically, after reconstitution of the peptides in TMT buffer, the individual samples were labeled using different TMT labels. After mixing, the samples were desalted on SEP-PAK C18 and fractionated into 15 fractions by high-pH reverse-phase chromatography. The individual fractions were vacuum-dried and stored at -80 °C.

### LC-MS/MS analysis

LC-MS/MS was performed using an Orbitrap Exploris 480 mass spectrometer coupled with an Easy-nLC 1200 system. After loading through the auto-sampler, the peptides were separated on a C18 analytical column (75 μm × 25 cm, C18, 1.9 μm, 100Å). The gradient was set up with mobile phase A (0.1% formic acid) and mobile phase B (80% ACN, 0.1% formic acid) and a flow rate of 300 nL/min. The DDA mode consisted of one full-scan mass spectrum (R = 60 K, AGC = 300%, max IT = 20 ms, scan range = 350–1500 *m/z*) followed by 20 MS/MS events (R = 15 K, AGC = 100%, max IT = auto, cycle time = 2 s, TurboTMT enabled). The HCD collision energy was set to 35, with a 1.2 Da isolation window and 35-s target ion exclusion.

### Database search

The raw MS data were analyzed with MaxQuant v1.6.6 using the Andromeda database search algorithm. The spectra files were searched against the UniProt.Proteome.Chicken.20,201,018.fasta database using the following parameters: TMT mode was checked for quantification; Variable modifications, Oxidation (M), Acetyl (Protein N-term) & Deamidation (NQ) ; Fixed modifications, Carbamidomethyl (C); Digestion, Trypsin/P; The MS1 match tolerance was set as 20 ppm for the first search and 4.5 ppm for the main search; the MS2 tolerance was set as 20 ppm. The results were filtered using an FDR of 1% for both proteins and peptides. After removal of decoy hits, contaminants, or proteins only identified by sites, the identified proteins were used for further analysis.

### Bioinformatics analysis

The identified DEPs were submitted to GO (http://geneontology.org/) and KEGG (http://www.genome.jp/kegg/) for annotation and pathway analysis respectively [86–88], using a threshold of *P* < 0.05 to indicate significant enrichment. Subcellular localization and upregulation and downregulation numbers were predicted using PSORTb and WoLF PSORT. Protein-protein interaction (PPI) networks were constructed by STRING (http://string-db.org/), and used k-means method to further cluster analysis (the number clusters: 3).

### Statistical analysis

GraphPad Prism (Version 9.0, San Diego, USA) was used for analysis and the compilation of graphs. Differences between two groups were determined by t-tests, the difference between LPS group and NC group was represented by #, and # *P* < 0.001, the difference between LPS-Andro group and LPS group was represented by *, and * *P* < 0.05, ** *P* < 0.01, and *** *P* < 0.001.

### Electronic supplementary material

Below is the link to the electronic supplementary material.

Supplementary Material 1

Supplementary Material 2

## Data Availability

The mass spectrometry proteomics data have been deposited to the ProteomeXchange Consortium (http://proteomecentral.proteomexchange.org) via the iProX partner repository with the dataset identifier PXD037079.
